# Le diagnostic anténatal de l'iniencéphalie

**DOI:** 10.11604/pamj.2015.20.194.2557

**Published:** 2015-03-03

**Authors:** Hanane Saadi, Abdelaziz Banani

**Affiliations:** 1 Service de Gynécologie Obstétrique I, CHU Hassan II, Fès, Maroc

**Keywords:** Iniencéphalie, diagnostic anténatal, malformation, Iniencephaly, ante-natal diagnosis, malformation

## Image en medicine

L'iniencéphalie est une malformation rare de la charnière cervico-occipitale. Elle appartient au spectre des anomalies du tube neural. C'est une malformation létale qui touche surtout le sexe féminin. Le diagnostic anténatal par échographie est possible dés 12 à 13 semaines d'aménorrhées (SA). L'iniencéphalie est caractérisée par une extension majeure de la tête, la continuité entre la tête et le tronc et un raccourcissement du rachis et les vertèbres cervicales avec non fermeture de l'arc postérieur. Elle peut être associée à d'autres malformations types une omphalocèle. Il n'y a pas d'indication d'une amniocentèse car il y a absence d'anomalie chromosomique. Le pronostic est sombre puisque elle est létale. Une interruption médicale de grossesse est indiquée une fois le diagnostic est posé. Un conseil génétique sera demandé et une prévention par acide folique sera proposée avant une prochaine grossesse. Nous rapportons l'observation d'une parturiente de 30 ans, grande multipare sans antécédents pathologiques notables. L’échographie de 22SA a révélé une encéphalocéle, une continuité entre la tête et le tronc et un pied bot. L'IRM fœtale a confirmé l'encéphalocéle et le pied bot et a montré une rachischisis. Une interruption médicale de grossesse a été réalisée.

**Figure 1:**
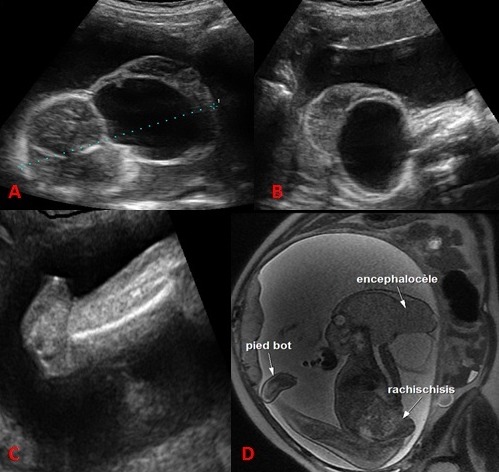
A) échographie anténatale de 22SA a montré une encéphalocéle; B) une continuité entre la tête et le tronc; C) un pied bot; D) L'IRM fœtale a confirmé ces anomalies avec une rachischisis

